# Training interval, feedback, and retention in cardiopulmonary resuscitation education: a systematic review

**DOI:** 10.1186/s12909-026-09538-4

**Published:** 2026-05-25

**Authors:** Hyeon-Ji Lee

**Affiliations:** Department of Paramedicine, College of Health Sciences, Kangwon National University, Samcheok, Republic of Korea

**Keywords:** Cardiopulmonary resuscitation, CPR education, Skill retention, Spaced training, Feedback mechanisms, Basic life support, Educational strategies, Systematic review

## Abstract

**Background:**

High-quality cardiopulmonary resuscitation (CPR) is a key determinant of survival after cardiac arrest; however, CPR skills deteriorate rapidly after training. Increasing attention has therefore been directed toward optimizing training design, including training intervals, feedback mechanisms, and educational delivery models, to improve skill retention. This systematic review aimed to synthesize evidence on the effectiveness of CPR education strategies, with particular emphasis on retention-oriented approaches.

**Methods:**

This systematic review was conducted in accordance with the PRISMA 2020 guidelines. A comprehensive literature search was performed in PubMed/MEDLINE, Embase, and the Cochrane Library for studies published from January 2015 to June 30, 2024. Interventional studies evaluating CPR education—including randomized controlled trials and quasi-experimental designs—were included. Due to substantial heterogeneity in study designs, interventions, and outcome measures, a meta-analysis was not feasible; therefore, a narrative synthesis was conducted.

**Results:**

A total of 20 studies were included. Across the included studies, spaced training was generally associated with improved skill retention compared with single-session training. Interventions incorporating feedback mechanisms were associated with improved compression performance, while reflective and cognitively oriented approaches showed potential for enhancing long-term skill retention, particularly at follow-up assessments. In addition, school- and community-based CPR education—including peer-led training—demonstrated outcomes comparable to traditional instructor-led models.

**Conclusions:**

These findings suggest that the effectiveness of CPR education is associated less with total training duration and more with how training is structured and reinforced. Retention-focused approaches—such as spaced training, feedback integration, and cognitively informed strategies—may support improved skill maintenance. However, given the heterogeneity of included studies and the presence of moderate to high risk of bias in several studies, these findings should be interpreted with caution.

## Introduction

Cardiac arrest remains a leading cause of mortality worldwide, and early bystander cardiopulmonary resuscitation (CPR) and the use of automated external defibrillators (AEDs) are well-established determinants of survival. Despite this, both the rate and quality of CPR performance in real-world settings often fall below recommended standards. Critical performance components—such as compression depth, rate, minimization of interruptions, and adherence to procedural sequence—have been shown to deteriorate rapidly following training, a phenomenon commonly described as skill decay [1]. Earlier studies conducted prior to the implementation of contemporary CPR guidelines similarly demonstrated substantial skill decay over time, even among trained providers [2–4]. However, given the substantial evolution in CPR training protocols and educational strategies over the past decade, the applicability of these earlier findings to current training contexts may be limited. Therefore, the effectiveness of CPR education depends not only on initial training but also on how well these skills are maintained over time.

Traditional CPR education has typically relied on single-session, instructor-led courses that combine didactic lectures with brief hands-on practice. While such approaches may improve immediate post-training performance, their effects tend to diminish without reinforcement [1, 5]. In recent years, increasing attention has been directed toward training interval and spaced practice, shifting the focus from short-term performance gains to sustained skill retention. Evidence indicates that, even when total training time is equivalent, shorter and repeated training sessions—or schedules tailored to individual performance—are associated with improved long-term skill maintenance [5]. Taken together, these observations highlight the need to view CPR education as an ongoing, reinforcement-based process rather than a one-time intervention.

In addition to training frequency, instructional modality and feedback mechanisms play a central role in determining educational effectiveness. Studies have consistently demonstrated that practice-oriented and demonstration-based approaches are associated with improved CPR performance compared with lecture-centered instruction [6]. Moreover, key CPR parameters—such as compression depth, rate, and recoil—are difficult for learners to evaluate independently, making real-time feedback essential for effective skill acquisition [7, 8]. Interventions incorporating feedback-enabled manikins or structured instructor feedback have therefore been widely explored as strategies to enhance both accuracy and retention of CPR skills [6–9].

Beyond technical performance, CPR requires the integration of cognitive, psychological, and team-based competencies. In actual cardiac arrest situations, factors such as stress, cognitive overload, and communication challenges may compromise performance despite prior training. To address these issues, recent educational approaches have incorporated elements such as reflective practice, structured team communication (e.g., TeamSTEPPS), and pacing strategies. These approaches aim not only to improve technical proficiency but also to support performance under realistic conditions [10–12].

At the same time, CPR education is increasingly being extended beyond healthcare professionals to broader populations. School-based basic life support (BLS) programs involving teachers, students, and peer instructors have demonstrated advantages in scalability and repeated exposure. Early and repeated training across the lifespan has been proposed as a key strategy for strengthening the chain of survival at the population level [13–15]. This shift underscores the importance of considering CPR education not only as a clinical competency but also as a public health intervention.

Several systematic reviews have previously examined CPR education and skill retention, including the work by Yeung et al. [16], which highlighted the potential benefits of feedback-enhanced and spaced training approaches. However, prior systematic reviews have primarily focused on isolated components of CPR education or specific populations, rather than providing an integrated synthesis across multiple educational domains within a single analytical framework. Consequently, a comprehensive synthesis focusing on retention-oriented training strategies across diverse educational domains remains limited. Furthermore, the growing body of recent interventional studies has not been comprehensively synthesized with a specific focus on retention-oriented training strategies. In additon, given the substantial evolution of CPR training guidelines and educational technologies over the past decade, there is a need to synthesize evidence that reflects contemporary training practices. Accordingly, this review focuses on studies published from 2015 onward, corresponding to the implementation of updated CPR training guidelines.

Building on this rationale, this systematic review aims to synthesize evidence from randomized controlled trials and interventional studies to examine four key aspects of CPR education: (1) training interval and spaced practice; (2) instructional modality and feedback provision; (3) integration of cognitive and psychological components; and (4) expansion of target populations and delivery models. By adopting an integrated perspective, this review seeks to identify key design elements and evidence-informed strategies for improving long-term CPR skill retention in contemporary training contexts.

## Methods

### Study design

This study was conducted as a systematic review in accordance with the PRISMA 2020 guidelines. The review focused on interventional studies evaluating cardiopulmonary resuscitation (CPR) education, with particular emphasis on skill retention and training design. Because the included studies showed substantial heterogeneity in outcome measures and follow-up timepoints, a predefined thematic narrative synthesis was adopted. Four themes were established a priori based on prior systematic reviews, the conceptual framework of CPR education, and commonly reported domains in the literature: (1) training interval and spaced practice; (2) instructional modality and feedback mechanisms; (3) cognitive, psychological, and team-based components; and (4) target populations and educational delivery models. This review was not prospectively registered in PROSPERO; however, the review protocol was developed in advance and the study was conducted in accordance with PRISMA 2020 guidelines.

### Search strategy

A comprehensive literature search was conducted in PubMed/MEDLINE, Embase, and the Cochrane Library for studies published from January 2015 to June 30, 2024. The start date was selected to align with the introduction of updated CPR training guidelines, which substantially influenced training protocols and educational interventions. Both Medical Subject Headings (MeSH) and free-text keywords were used. The primary search strategy included the following terms:

(“cardiopulmonary resuscitation” OR “CPR” OR “basic life support” OR “BLS”)

AND

(“training” OR “education” OR “instruction” OR “teaching”)

AND

(“retention” OR “skill decay” OR “training interval” OR “spaced practice” OR “feedback” OR “peer education”)

Search results were imported into reference management software, and duplicate records were removed. In addition, the reference lists of included studies and relevant review articles were manually screened to identify additional eligible studies.

### Eligibility criteria

Studies were included if they met the following criteria:

1. evaluated CPR educational interventions;
2. compared at least one of the following components: training interval, instructional modality, feedback provision, cognitive–psychological or team-based elements, or target population;
3. used a randomized controlled, quasi-experimental, or other interventional design;
4. were published in English; and
5. were available in full text.

Studies were excluded if they were case reports, narrative reviews, meta-analyses, editorials, studies without an educational intervention (e.g., perception-only surveys), studies not reporting CPR performance or skill retention outcomes, abstract-only publications, or studies published prior to January 2015, as this review aimed to reflect contemporary CPR training guidelines and educational practices.

### Study selection process

**Fig. 1:**
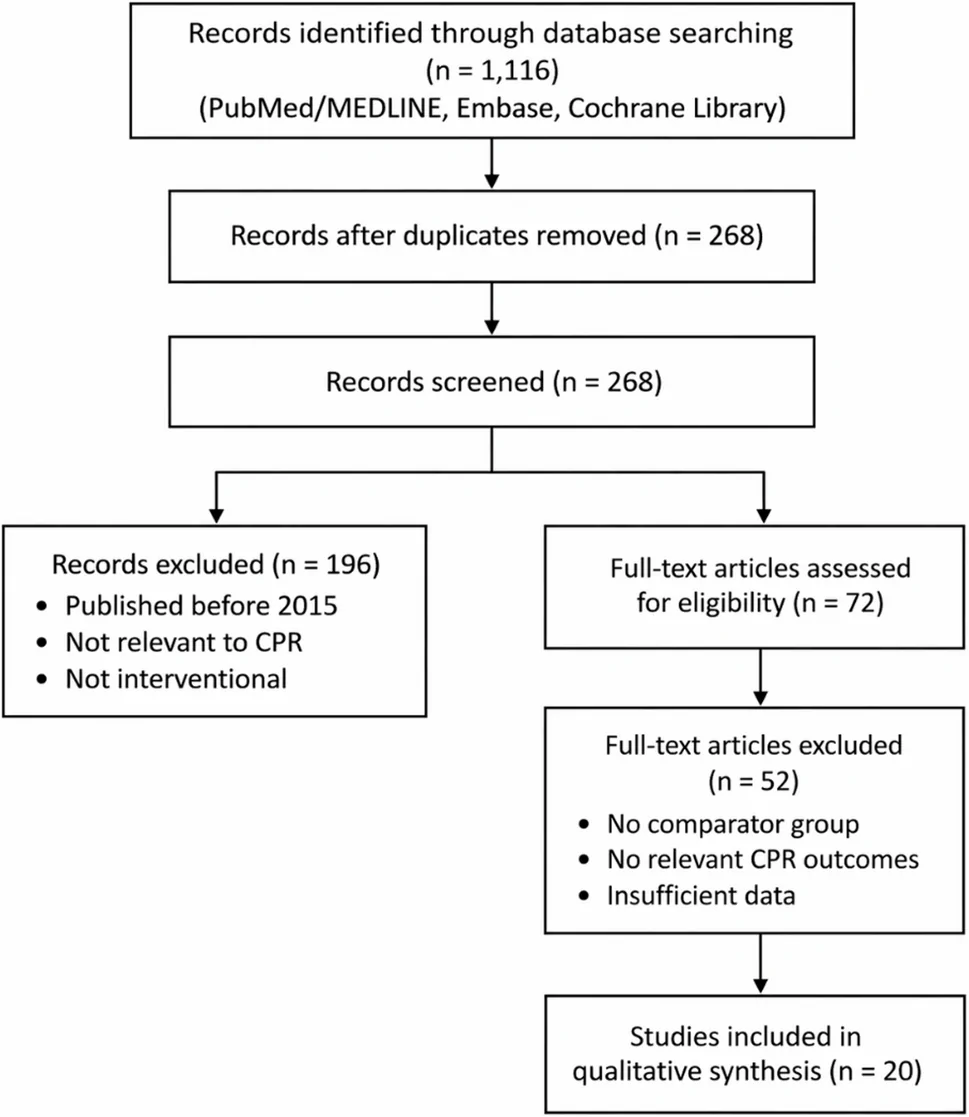
PRISMA 2020 flow diagram illustrating the study selection process for the systematic review of cardiopulmonary resuscitation education interventions. Studies published prior to 2015 were excluded during the screening process based on the predefined inclusion criteria

Study selection (title and abstract screening followed by full-text review) was conducted by the author in accordance with the predefined inclusion and exclusion criteria. To enhance methodological rigor, a trained research assistant independently reviewed a subset of the screening decisions. Any discrepancies were discussed and resolved through consensus. The search identified 1,116 records. After removing duplicates, titles and abstracts were screened, during which studies published prior to 2015 were excluded based on the predefined eligibility criteria. A total of 72 full-text articles were assessed for eligibility, of which 52 were excluded for reasons including insufficient description of the educational intervention, absence of a comparator group, outcomes not directly related to CPR performance or retention, or inability to extract relevant data. Ultimately, 20 studies met all inclusion criteria and were included in the qualitative synthesis. The study selection process is illustrated in Fig. 1.

### Data extraction and synthesis

Data extraction was performed by the author using a standardized extraction form. The extracted items included author and publication year, study design and country, participant characteristics, details of the educational intervention, comparator conditions, and outcomes related to CPR performance, skill retention, and cognitive or psychological measures.

To ensure accuracy and reliability, a trained research assistant independently verified a subset of the extracted data. Any discrepancies were discussed and resolved through consensus, with final decisions made by the author. Due to substantial heterogeneity in study design, interventions, and outcome measures, meta-analysis was not performed. Instead, findings were synthesized narratively according to the four predefined themes. Representative randomized controlled trials reporting quantitative outcomes and between-group comparisons are summarized in the Results section to illustrate key findings related to CPR skill retention. 

### Risk of bias and study quality assessment

Methodological quality was assessed according to study design. Randomized controlled trials were evaluated using the Cochrane Risk of Bias 2 (RoB 2) tool, whereas non-randomized studies were assessed using the ROBINS-I tool. The assessed domains included the randomization process, deviations from intended interventions, missing outcome data, outcome measurement, and selective reporting. Risk-of-bias assessment was conducted by the author. To enhance reliability, a trained research assistant independently reviewed a subset of the assessments. Any discrepancies were discussed and resolved through consensus, with final decisions made by the author.

## Results

### Study characteristics

A total of 20 studies were included in this systematic review. The main characteristics of the included studies—including country, study design, participants, intervention, comparator, and key outcomes—are summarized in Table 1.

**Table 1:** Characteristics of studies included in the systematic review

No.	Author (Year)	Country	Study Design	Participants	Intervention / Comparator	Key Outcome
1	Li et al. (2015) [17]	China	Non-randomized	Students	3D CPR game	Improved skill retention
2	Beck et al. (2015) [14]	Germany	RCT	School students	Peer vs. instructor-led CPR	Non-inferior performance compared to instructor-led training
3	Charlier et al. (2016) [18]	Belgium	Non-randomized	Students	Jigsaw peer learning	Improved CPR skills
4	García del Águila et al. (2019) [13]	Spain	Non-randomized	Teachers	School-based BLS training	Improved CPR knowledge and skills
5	Fukushima et al. (2019) [11]	Japan	Non-randomized	Healthcare teams	TeamSTEPPS-based training	Improved psychological outcomes
6	Rodriguez-Ruiz et al. (2019) [19]	Spain	Non-randomized	Healthcare providers	New infant CPR technique	Improved compression quality
7	Smereka et al. (2019) [20]	Poland	Non-randomized	Dental hygienists	Emergency response training	Improved preparedness
8	Kneba & Humm (2020) [12]	USA	Non-randomized	Veterinary students	Mental metronome use	Improved compression rate
9	Oermann et al. (2020) [1]	USA	RCT	Nursing students	Different CPR training intervals	Improved skill retention with spaced training
10	Suet et al. (2020) [7]	France	RCT	Medical students	Feedback vs. standard CPR training	Improved compression performance with feedback
11	Hansen et al. (2020) [6]	Denmark	RCT	Laypersons	Demonstration vs. lecture	Improved compression performance with demonstration
12	Martínez-Isasi et al. (2021) [21]	Spain	Non-randomized	School nurses/students	Repeated school CPR training	Improved long-term retention
13	Marcus et al. (2022) [9]	Germany	RCT	Medical students	Video vs. instructor-led CPR	Comparable skill acquisition
14	Oermann et al. (2022) [5]	USA	RCT	Nursing students	Personalized CPR schedules	Improved skill retention
15	Thommes et al. (2023) [10]	Germany	RCT	Laypersons	Reflective practice CPR training	Improved long-term skill retention
16	Chang et al. (2023) [8]	Taiwan	RCT	University students	Different CPR education methods	Improved CPR performance
17	Pedrazas-López et al. (2023) [22]	Spain	RCT	Preschool children	RCParvulari program	Improved early CPR learning
18	McGlinchey Ford et al. (2024) [23]	USA	RCT	Elementary students	CPR education program	Improved CPR skill acquisition
19	Stirparo et al. (2024) [24]	Italy	Non-randomized	Physicians	Practical autonomy training	Improved self-confidence
20	Spartinou et al. (2024) [15]	Greece	Non-randomized	Secondary students	Peer education CPR training	Improved skill retention

The majority of included studies were randomized controlled trials, along with several quasi-experimental and observational designs. Study populations were diverse and included healthcare professionals, medical and nursing students, school-aged children, and laypersons. Interventions varied in terms of training interval, instructional modality, feedback provision, and incorporation of cognitive or psychological components. The majority of randomized controlled trials were conducted in educational settings, whereas non-randomized studies were more frequently applied in real-world or community-based contexts.

### Risk of bias assessment

The methodological quality of the included studies varied across study designs. Among the randomized controlled trials assessed using the RoB 2 tool, several studies were judged to have some concerns, primarily due to limitations in blinding and incomplete outcome reporting. A smaller number of studies were rated as low risk of bias. For non-randomized studies assessed using the ROBINS-I tool, the overall risk of bias was generally moderate, with common concerns related to confounding, selection bias, and limitations in outcome measurement. No studies were classified as having serious or critical risk of bias. Overall, the distribution of risk-of-bias judgments indicates variability in study quality across the included literature, which should be considered when interpreting the findings of this review. A summary of the risk-of-bias assessment is presented in Table 2.

**Table 2:** Summary of risk-of-bias assessment of included studies

Study	Study design	Assessment tool	Risk of bias
Li et al. (2015) [17]	Non-randomized	ROBINS-I	Moderate
Beck et al. (2015) [14]	RCT	RoB 2	Some concerns
Charlier et al. (2016) [18]	Non-randomized	ROBINS-I	Moderate
García del Águila et al. (2019) [13]	Non-randomized	ROBINS-I	Moderate
Fukushima et al. (2019) [11]	Non-randomized	ROBINS-I	Moderate
Rodriguez-Ruiz et al. (2019) [19]	Non-randomized	ROBINS-I	Moderate
Smereka et al. (2019) [20]	Non-randomized	ROBINS-I	Moderate
Kneba & Humm (2020) [12]	Non-randomized	ROBINS-I	Moderate
Oermann et al. (2020) [1]	RCT	RoB 2	Low risk
Suet et al. (2020) [7]	RCT	RoB 2	Some concerns
Hansen et al. (2020) [6]	RCT	RoB 2	Low risk
Martínez-Isasi et al. (2021) [21]	Non-randomized	ROBINS-I	Moderate
Marcus et al. (2022) [9]	RCT	RoB 2	Some concerns
Oermann et al. (2022) [5]	RCT	RoB 2	Low risk
Thommes et al. (2023) [10]	RCT	RoB 2	Some concerns
Chang et al. (2023) [8]	RCT	RoB 2	Some concerns
Pedrazas-López et al. (2023) [22]	RCT	RoB 2	Some concerns
McGlinchey Ford et al. (2024) [23]	RCT	RoB 2	Some concerns
Stirparo et al. (2024) [24]	Non-randomized	ROBINS-I	Moderate
Spartinou et al. (2024) [15]	Non-randomized	ROBINS-I	Moderate

### Instructional modality and feedback mechanisms

Studies examining instructional modality and feedback mechanisms generally showed that active, feedback-oriented training approaches were associated with improved CPR performance. Across randomized trials, interventions incorporating real-time feedback demonstrated more accurate chest compression performance compared with standard instruction. This pattern likely reflects the difficulty of self-assessing key CPR parameters, such as compression depth and rate, without external guidance. Similarly, demonstration-based training tended to result in more accurate performance than lecture-based approaches, suggesting that observational learning combined with practice facilitates more effective skill acquisition. Although video-based instruction achieved comparable immediate knowledge outcomes, interventions that incorporated hands-on practice and feedback were more likely to support sustained performance at follow-up. Table 3 presents representative randomized controlled trials illustrating these patterns, including comparisons of spaced versus massed training, feedback-based versus standard instruction, and reflective versus conventional training approaches.

**Table 3:** Summary of quantitative outcomes from representative randomized controlled trials evaluating CPR education interventions. Multiple entries from the same study represent different outcome measures or follow-up timepoints

Study	Comparison	Outcome	Timepoint	Key finding	Retention
Beck (2015) [14]	Peer vs. instructor	CPR pass rate	Post	Peer training non-inferior to instructor-led	No
Oermann (2020) [1]	Spaced vs. massed	Compression depth	Post	Spaced training showed improved performance	No
Oermann (2020) [1]	Spaced vs. massed	Compression depth	3 months	Better retention with spaced training	Yes
Suet (2020) [7]	Feedback vs. standard	Compression accuracy	Post	Higher accuracy with feedback-based training	No
Hansen (2020) [6]	Demonstration vs. lecture	Compression rate	Post	Demonstration-based training superior	No
Thommes (2023) [10]	Reflective vs. standard	BLS skill score	6 months	Improved long-term retention with reflective practice	Yes

### Cognitive–psychological and reflective training components

Several studies evaluated the impact of cognitive and psychological components on CPR training outcomes. Reflective practice–based interventions were associated with improved long-term skill retention, particularly at follow-up assessments. These approaches emphasize structured self-assessment and performance reflection, which may enhance consolidation of both cognitive and motor skills. Team-based training interventions incorporating structured communication strategies were linked to improvements in psychological outcomes, including confidence and role clarity. These findings suggest that effective CPR performance depends not only on technical proficiency but also on coordination and decision-making under pressure. In addition, the use of rhythm or pacing aids was associated with more consistent compression rates, likely by reducing cognitive load during task execution.

Taken together, these results indicate that integrating cognitive and psychological elements into CPR training may improve both performance stability and retention.

### Expansion of target populations and training delivery

Studies extending CPR education to non-medical populations demonstrated that effective training can be achieved across diverse learner groups. School-based programs involving teachers, students, and peer instructors showed performance outcomes comparable to traditional instructor-led models. In particular, randomized trials reported that peer-led training was non-inferior to instructor-led approaches, supporting the scalability of alternative training models. Studies involving younger populations, including elementary and preschool-aged children, indicated that age-appropriate instructional strategies and repeated exposure enabled successful acquisition of basic CPR skills.

These findings suggest that CPR education can be effectively adapted to a wide range of populations, provided that training is tailored to learner characteristics and reinforced over time.

## Discussion

This systematic review synthesized evidence on key determinants of effective CPR education across four domains: training interval, instructional modality and feedback mechanisms, integration of cognitive and psychological components, and expansion of target populations.

Overall, the findings indicate that CPR education outcomes are influenced less by total training duration alone and more by how training is structured, reinforced, and sustained over time. However, these findings should be interpreted with caution due to variability in study design, outcome measures, and risk of bias across the included studies.

### Importance of training interval and skill retention

A consistent pattern across included studies was that spaced training was associated with improved CPR skill retention compared with single-session, massed instruction. Several studies reported rapid skill decay following one-time training despite initial performance gains, whereas repeated or spaced training was associated with more sustained performance over time [1, 14]. This pattern is consistent with established learning theory, in which distributed practice enhances memory consolidation and reduces skill decay compared with massed learning. This observation is consistent with earlier foundational studies conducted prior to 2015, which also reported rapid deterioration of CPR skills in the absence of periodic retraining [2–4]. However, these earlier studies were conducted under previous CPR guideline frameworks, and differences in training protocols and educational technologies may limit direct comparison with contemporary interventions. In the context of CPR, where both psychomotor performance and decision-making must be retained under stress, periodic reinforcement may be particularly important [1, 5]. However, it should be noted that many studies assessed retention over relatively short follow-up periods, and the long-term durability of these effects remains uncertain. In addition, several included studies demonstrated moderate to high risk of bias, which may affect the strength of the observed associations. Therefore, the observed associations should be interpreted in light of potential methodological limitations.

### Teaching modality and the role of feedback

Instructional modality and feedback mechanisms were also consistently associated with improved CPR performance outcomes. Interventions incorporating hands-on practice and real-time feedback were generally associated with better performance compared with lecture-based approaches [8, 15, 20, 24]. One explanation is that CPR performance parameters—such as compression depth and rate—are difficult for learners to self-assess accurately. Without feedback, incorrect techniques may persist despite repeated practice. In contrast, feedback-enabled training allows learners to recognize and correct errors in real time, supporting more accurate skill acquisition [8, 20]. Nevertheless, the magnitude of these effects varied across studies, and differences in study design, measurement tools, and training environments limit direct comparison. As such, these findings should be interpreted as indicative rather than definitive evidence.

### Integration of cognitive, psychological, and reflective components

CPR performance involves not only psychomotor execution but also cognitive processing, emotional regulation, and team coordination. Several studies that incorporated cognitive and psychological components—such as reflective practice and team-based training—reported improved performance or retention outcomes [6, 10, 11, 13].

These findings can be understood in the context of cognitive load theory and stress-related performance limitations. In real cardiac arrest scenarios, anxiety, cognitive overload, and communication challenges may impair performance even among trained individuals. Interventions addressing these factors may help reduce the gap between simulated training and real-world performance [10–12]. However, the number of studies in this domain remains limited, and variability in intervention design and outcome measures restricts the strength of conclusions that can be drawn.

### Expansion of target populations and training delivery models

The included studies demonstrated that CPR education can be extended beyond healthcare professionals to broader populations. School- and community-based programs showed meaningful skill acquisition and retention, with some studies reporting comparable outcomes between peer-led and instructor-led training models [5, 7, 18, 19, 21–23]. These findings are further supported by additional studies included in this review that evaluated diverse training populations and educational approaches [17, 18]. However, differences in participant characteristics, baseline skills, and training contexts limit generalizability. Further research is needed to determine how these findings translate into real-world bystander CPR performance.

### Implications for CPR education and future research

Overall, the findings of this review suggest that CPR education effectiveness is associated with the integration of multiple factors, including training interval design, feedback mechanisms, and cognitive–psychological components, consistent with prior evidence in the literature [1, 5, 8, 16]. Importantly, this review focused on studies published from 2015 onward to reflect contemporary CPR training guidelines and educational practices. As CPR protocols and training technologies have evolved substantially over time, earlier studies may not be directly comparable to current educational approaches. Future research should move beyond evaluating isolated educational elements and instead investigate integrated training approaches that combine these components. In particular, studies examining long-term retention, real-world performance, and implementation in community settings are needed to strengthen the evidence base.

## Limitations

This systematic review has several limitations that should be considered when interpreting the findings.

First, substantial heterogeneity existed among the included studies with respect to study design, participant characteristics, educational settings, and outcome measures. As a result, quantitative meta-analysis was not feasible, and a narrative synthesis approach was adopted.

Second, outcome measures and follow-up timepoints varied considerably across studies. While some studies evaluated CPR performance immediately after training, relatively few assessed long-term retention. Therefore, conclusions regarding retention should be interpreted with caution.

Third, the methodological quality of included studies varied, with several studies demonstrating moderate to high risk of bias. This may affect the reliability of observed associations between educational interventions and outcomes.

Fourth, this review included only studies published from 2015 onward to reflect contemporary CPR guidelines. While this approach improves relevance to current practice, it may have excluded earlier studies that could provide additional historical context.

Fifth, only English-language studies were included, and gray literature was not systematically searched, which may introduce language and publication bias.

Finally, the selective inclusion of representative studies in Table 3 may introduce reporting bias, although this approach was used to enhance interpretability.

## Conclusions

This systematic review suggests that the effectiveness of CPR education is associated less with total training duration and more with how training is structured, reinforced, and sustained over time. Spaced training and feedback-based instructional approaches were consistently associated with improved CPR performance and retention across included studies. In addition, educational strategies incorporating cognitive and reflective components, as well as alternative delivery models such as peer-led training, showed promising results across diverse learner populations.

However, given the heterogeneity of included studies, variability in outcome measures, and the presence of moderate to high risk of bias in several studies, these findings should be interpreted with caution.

Future research should focus on integrated training models that combine optimized training intervals, feedback mechanisms, and cognitive–behavioral components, with particular emphasis on long-term retention and real-world implementation.

## Data Availability

All data generated or analyzed during this study are included in this published article. The search strategy, study selection numbers (PRISMA flow), and characteristics of included studies are reported in the main text and Table 1-2.
